# Neutrophil-lymphocyte ratio is associated with arterial stiffness in patients with peritoneal dialysis

**DOI:** 10.1186/s12882-016-0394-4

**Published:** 2016-11-24

**Authors:** Kedan Cai, Qun Luo, Beixia Zhu, Lina Han, Dan Wu, Zhiwei Dai, Kaiyue Wang

**Affiliations:** Department of Nephrology, Ningbo No. 2 Hospital, Ningbo University School of Medicine, Zhejiang, China

**Keywords:** Neutrophil-lymphocyte ratio, Inflammation, Arterial stiffness, Brachial-ankle pulse wave velocity, Peritoneal dialysis

## Abstract

**Background:**

Patients with peritoneal dialysis are in the persistent inflammation state and have elevated arterial stiffness. Neutrophil-lymphocyte ratio(NLR) is a new inflammatory marker in renal and cardiac disorders. Brachial-ankle pulse wave velocity (baPWV) is a non-invasive measurement, which is widely used as a surrogate marker of arterial stiffness. However, there is little evidence to show an association between NLR and baPWV in patients with peritoneal dialysis. The aim of this cross-section study was to investigate the relationship between NLR and arterial stiffness measured by baPWV in patients with peritoneal dialysis.

**Methods:**

In this cross-section study, 101 patients with peritoneal dialysis were enrolled from January 2014 to June 2015. According to average baPWV level (1847.54 cm/s), the patients were categorized into two groups, low group and high group. baPWV, which reflects arterial stiffness, was calculated using the single-point method. Clinical data were collected in details. NLR was calculated using complete blood count. Associations between NLR and baPWV were assessed using Pearson’s correlation and linear regression analysis.

**Results:**

The NLR was significantly lower in the low baPWV group than in the high baPWV group (*p* = 0.03). There were positive correlations between baPWV and neutrophil count (*r* = 0.24, *p* = 0.01) and NRL(*r* = 0.43, *P* < 0.01), and there was a negative correlation between baPWV and lymphocyte count (*r* = -0.23, *p* = 0.01). In addition, albumin, phosphorous and intact parathyroid hormone showed negative correlations with baPWV (*r* = −0.32, *p* < 0.01; *r* = −0.28, *p* < 0.01; *r* = −0.25, *p* = 0.01, respectively). Age and hsCRP showed positive correlations with baPWV (*r* = 0.47, *p* < 0.01; *r* = 0.25, *p* = 0.01). In multivariate analysis, NLR independently correlated with baPWV in patients with peritoneal dialysis (β = 0.33, *p* < 0.01), even after adjustment for various confounders.

**Conclusion:**

Our study suggests that NLR was an independently associated with arterial stiffness in patients with peritoneal dialysis. However, further prospective studies are needed to confirm cause-and-effect relationship between NLR and baPWV, and to investigate whether anti-inflammatory treatment could improve arterial stiffness in patients with peritoneal dialysis.

**Electronic supplementary material:**

The online version of this article (doi:10.1186/s12882-016-0394-4) contains supplementary material, which is available to authorized users.

## Background

In 2014, 55,373 patients received peritoneal dialysis (PD) in China. Cardiovascular disease is the first etiology of mortality and morbidity, accounting for nearly 60% of all deaths in the patients with PD [1]. Microinflammation is a key component of the malnutrition- inflammation-atherosclerosis and calcification syndrome (MIAC syndrome), which is associated with increased risk of cardiovascular disease in patients with PD.

Recently, neutrophil-to-lymphocyte ratio (NLR), calculated as a ratio of neutrophil to lymphocyte in peripheral blood, is regarded a readily available indicator for the severity and extension of systemic inflammation and atherosclerosis in renal and cardiac disorders [2, 3].

Elevated arterial stiffness is an early marker of systemic atherosclerosis, which has been shown to be a powerful independent predictor of cardiovascular events and all-cause mortality in chronic kidney disease(CKD) [4]. Pulse wave velocity (PWV) is a non-invasive measurement, which is widely used as a surrogate marker of arterial stiffness [5]. Brachial-ankle PWV (baPWV) correlates with the gold standard measurement, carotid-femoral PWV [6]. Increasing studies demonstrated that elevated baPWV is associated with increased risk of renal disease and cardiovascular diseases, as well as increased total mortality [7, 8].

However, there is little evidence to show an association between NLR and baPWV in patients with PD. Therefore, we aimed to evaluate the relationship between NLR and baPWV in patients with PD.

## Methods

### Patients

**Fig 1.**
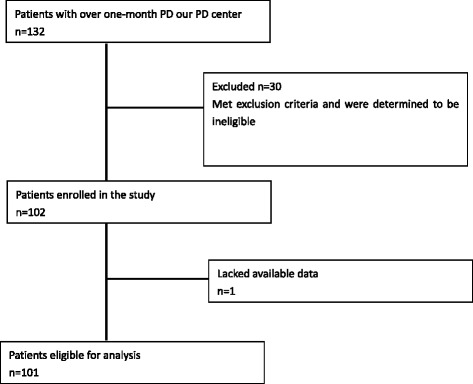
Patient selection scheme

Patients with over one-month PD were included in this cross-section study from January 2014 to June 2015 in Ningbo No.2 Hospital. The number of cases during the study period determined the sample size. It has adhered to the STROBE guidelines for observational studies. According to average baPWV level (1847.54 cm/s), the patients were categorized into two groups, low baPWV and high baPWV. Patients with active infection, severe liver dysfunction, malignancy, hematological diseases were excluded. The flow diagram is shown in Fig. 1. Patients were dialyzed with dextrose peritoneal dialysate produced by Baxter Healthcare (Guangzhou, China). This study was approved by the Ethics Committee of Ningbo No.2 Hospital.

### Demographic data and biochemical measurements

The demographic data included age, gender, etiology of end-stage renal disease, prevalence of diabetes, the dialysis duration, current smoking status, and atherosclerotic vascular disease (AVD) history. Physical examination included blood pressure, height, and weight. Blood sample was drawn in the morning following a fasting period of over 8 h. 24-hours ultrafiltration volume, and urine output volume were recorded. Biochemical measurements, using standard laboratory techniques in the same laboratory center, included blood cell counts, neutrophic counts, lymphocyte counts, haemoglobin, platelets, calcium, phosphorus, intact parathyroid hormone (iPTH), creatinine, urea nitrogen, total cholesterol, triglycerides, albumin and highly sensitive C-reactive protein (hsCRP). Complete blood counts were analysed using Sysmex XT-2000i (Sysmex Corporation). Serum calcium, phosphate, renal profiles, albumin hsCRP, and lipid profiles were measured using ADVIA 2400 Chemistry System(Siemens Healthcare Diagnostics). PTH levels were determined using ADVIA Centaur XP (Siemens Healthcare Diagnostics). All of these data were collected from Hospital Information System of Ningbo No.2 Hospital.

NLR was calculated as a ratio of neutrophil-to-lymphocyte in peripheral blood. Mean arterial pressure (MAP) was calculated as 1/3 systolic blood pressure plus 2/3 diastolic blood pressure. Body mass index (BMI) was calculated as weight (kg) divided by the square of height (m^2^). Residual renal function (RRF) was assessed by calculating the glomerular filtration rate (GFR) using the Chronic Kidney Disease Epidemiology Collaboration equation. Adequacy of dialysis (total Kt/V) was measured using PD Adequest software.

### Measurement of the brachial-ankle pulse wave velocity (baPWV)

baPWV was measured according to the method in a previous study [9], measurement of baPWV was performed after a 15-min rest in a supine position in a quiet and temperature-controlled room. The baPWV was measured based on regular methods using an Omron waveform analyzer (BP-203RPE III; Omron Co. Ltd, Dalian, China). The pulse wave velocity between the bilateral brachial and ankle artery was measured by placing both the arm and ankle in cuffs into which an oscillometric sensor was implanted. All the measurement were completed by the same doctor.

### Statistical analysis

All statistical analyses were performed with SPSS software, version 19.0 (SPSS Inc.USA). A *p*-value < 0.05 was considered statistically significant. The results were expressed as frequencies and percentages for categorical variables, means with standard deviation for normally distributed continuous variables, and median values (interquartile ranges) for non-normally distributed continuous variables, respectively. Student’s *t*-test for independent samples was used for normally distributed c ontinuous variables. Comparisons of non-normally distributed continuous variables were performed using the Mann-Whitney *U*-test. For categorical variables, the chi-square test was used. Correlations of baPWV with NRL and other variables were assessed using Pearson’s or Spearman’s correlation analysis. Univariate and multiple linear regression analyses were performed to identify the predictors of baPWV. Variables found to be statistically significant in univariate analyses were entered into multivariate lineal regression model.

## Results

The demographic and biochemical characteristics of 101 subjects, according to baPWV levels are shown in Table 1. There were 58 patients in the low baPWV group and 43 patients in the high group. The raw data of the study can be seen in more detail (see Additional file 1). There were 100 patients treated with CAPD modality and one patient treated with APD modality. The primary etiology of end stage renal disease (ESRD) was chronic glomerulonephritis in both groups. There were significantly more patients with diabetes mellitus in the high baPWV group compared to the low baPWV group (*x* ^2^ = 11.63, *p* < 0.01).The number of the patients with cardiovascular diseases and current smoking status were similar between both groups. There were no differences between the groups in term of the following parameters: BMI, peritoneal dialysis duration, ultrafiltration, urine output, hemoglobin and triglyceride. Levels of albumin, total cholesterol, phosphorous, iPTH in the low group were significantly lower than those in the high group (*p* < 0.01, *p* = 0.03, *p* = 0.01, *p* = 0.02, respectively). However, age and hsCRP were significantly higher in the low group than that in the high group (both *p* < 0.01). There were slight differences in MAP, calcium, and Kt/V between groups, without reaching statistical significance (*p* = 0.10, *p* = 0.07, *p* = 0.09, respectively). In sub-analysis, average of baPWV was higher in patients with AVD history than those without AVD history (t = -2.99, *p* < 0.01)

**Table 1:** Baseline demographic and clinical characteristics of PD patients stratified by average baPWV level

Variables	PWV			
	Low (*n* = 58)	High (*n* = 43)	T/u/*x* ^2^	*P*-value
Age (year)	48.60 ± 14.20	63.60 ± 12.65	−5.49	<0.01*
Gender (male, %)	30(51.72)	30(69.76)	3.33	0.06
Body mass index (kg/m^2^)	21.16 ± 3.03	20.81 ± 2.83	0.59	0.55
Peritoneal dialysis duration (months)			−0.05	0.95
Median	16.00	16.00		
Interquartile range	4.75,45.00	7.00,46.00		
Diabetes (%)	6(10.34)	13(30.23)	6.39	0.01*
Etiology of ESRD			5.59	0.06
Chronic glomerular nephritis (%)	44(75.86)	23(53.48)		
Diabetic kidney disease (%)	5(5.17)	8(18.60)		
Others(%)	9(15.51)	12(27.90)		
current smoking status(%)	5(8.62)	6(13.95)	0.72	0.39
cardiovascular disease history(%)	3(5.17)	6(13.95)	2.34	0.12
MAP (mmHg)	105.17 ± 17.69	110.93 ± 17.24	−1.63	0.10
Net UF (ml/day)			−1.02	0.30
Median	212.50	270.00		
Interquartile range	−50.00,712.50	70.00,720		
Urine output (ml/day)			−1.32	0.18
Median	600.00	500.00		
Interquartile range	200.00,1325.00	20.00,1000.00		
Hemoglobin (g/L)	104.17 ± 24.25	97.12 ± 18.20	1.66	0.10
Albumin (g/L)	36.18 ± 4.84	31.09 ± 5.70	4.83	<0.01*
Total cholesterol (mmol/L)	5.18 ± 1.21	4.64 ± 1.26	2.18	0.03*
Triglycerides (mmol/L)	1.78 ± 0.95	1.59 ± 1.02	0.96	0.33
Calcium (mmol/L)	2.10 ± 0.24	2.01 ± 0.23	1.80	0.07
Phosphorus (mmol/L)	1.60 ± 0.37	1.37 ± 0.49	2.64	0.01*
Intact parathyroid hormone (pg/ml)	434.62 ± 328.77	298.26 ± 240.22	2.30	0.02*
rGFR (ml/min/1.73 m^2^)	5.67 ± 2.68	6.29 ± 2.96	−1.09	0.27
Kt/V	2.11 ± 0.46	1.95 ± 0.42	1.71	0.09
hsCRP(mg/dl)			−2.90	<0.01*
Median	1.19	4.68		
Interquartile range	0.61,3.53	0.66,12.04		
WBC (*10^3/mm^3^)	6.47 ± 1.71	6.42 ± 2.11	0.13	0.89
Neutrophil (*10^3/mm^3^)	4.32 ± 1.35	4.41 ± 1.71	−0.30	0.76
Lymphocyte(*10^3/mm^3^)	1.45 ± 0.43	1.24 ± 0.42	2.45	0.01*
NLR	3.17 ± 1.20	3.89 ± 1.92	−2.15	0.03*
baPWV(cm/s)	1506.21 ± 188.69	2284.45 ± 508.79	−9.55	<0.01*

*MAP* mean arterial pressure, *UF* ultrafiltation, *GFR* glomerular filtration rate, *hsCRP* high-sensitive C-reactive protein, *WBC* white blood count, *baPWV* brachial-ankle pulse wave velocity, *NLR* neutrophil-lymphocyte ratio;*p < 0.05

When hematologic parameters were analyzed, it was observed that white blood cell (WBC) count and neutrophil counts were similar between groups (*p* = 0.89, *p* = 0.76, respectively). However, the lymphocyte count was significantly higher in low group compared to in high group (*p* = 0.01). NLR was significantly lower in the low group than that in the high group (*p* = 0.03) (Table 1).

Correlations between baPWV and parameters in patients with PD are shown in Table 2. According to the results, there were positive correlations of baPWV and neutrophil count (*r* = 0.24, *p* = 0.01) and NRL (*r* = 0.43, *p* < 0.01), and there was a negative correlation between baPWV and lymphocyte count (*r* = -0.23, *P* = 0.01). In addition, albumin, phosphorous and iPTH showed negative correlations with baPWV (*r* = -0.32, *P* < 0.01; *r* = -0.28, *P* < 0.01; *r* = -0.25, *P* = 0.01). Age and hsCRP showed positive correlation with baPWV (*r* = 0.47, *p* < 0.01; *r* = 0.25, *p* = 0.01). Correlation between baPWV and MAP approached statistical significance (*r* = 0.17, *p* = 0.08).

**Table 2:** Correlation analysis for variables and baPWV

Variables	*r*	*p*-value
Age (years)	0.47	<0.01*
Body mass index (kg/m^2^)	−0.11	0.25
Peritoneal dialysis duration (months)	−0.01	0.84
Diabetes	0.25	0.01*
current smoking status	0.23	0.01*
atherosclerotic vascular disease history	0.24	0.01*
MAP (mmHg)	0.17	0.08
Net UF (ml/day)	0.15	0.13
Urine output (ml/day)	0.15	0.11
Hemoglobin (g/L)	−0.15	0.12
Albumin (g/L)	−0.32	<0.01*
Total cholesterol (mmol/L)	−0.12	0.22
Triglycerides (mmol/L)	−0.09	0.34
Calcium (mmol/L)	−0.03	0.70
Phosphorus (mmol/L)	−0.28	<0.01*
Intact parathyroid hormone (pg/ml)	−0.25	0.01*
rGFR (ml/min/1.73 m^2^)	0.14	0.13
Kt/V	−0.15	0.12
hsCRP(mg/dl)	0.25	0.01*
WBC (*10^3/mm^3^)	0.16	0.09
Neutrophil (*10^3/mm^3^)	0.24	0.01*
Lymphocyte(*10^3/mm^3^)	−0.23	0.01*
NLR	0.43	<0.01*

*MAP* mean arterial pressure, *UF* ultrafiltation, *GFR* glomerular filtration rate, *hsCRP* high-sensitive C-reactive protein, *WBC* white blood count, *baPWV* brachial-ankle pulse wave velocity, *NLR* neutrophil-lymphocyte ratio;*p < 0.05

Variables found to be statistically significant in univariate analyses were entered into multivariate lineal regression model, age, diabetes, current smoking status, AVD history, albumin,phosphorus, iPTH and NLR. In multivariate analysis, NLR independently correlated with baPWV in patients with PD (β = 0.33, *p* < 0.01) (Table 3).

**Table 3:** Univariate and multivariate associates of baPWV in patients with peritoneal dialysis

Variables	baPWV					
	Univariable			Multivariate		
	β coefficient	t	*P*-value	β coefficient	t	*P*-value
Age (year)	0.47	5.38	<0.01*	0.25	2.61	<0.01*
Body mass index (kg/m^2^)	−0.11	−1.14	0.26	--	--	--
Diabetes (%)	0.23	2.44	0.01*	0.04	0.53	0.59
current smoking status (%)	0.25	2.55	0.01*	−0.18	−1.00	0.31
atherosclerotic vascular disease history (%)	0.28	2.99	<0.01*	0.32	1.75	0.08
MAP (mmHg)	−0.17	1.72	0.08	--	--	--
Albumin (g/L)	−0.31	−3.34	<0.01*	−0.17	−1.99	0.04*
Calcium (mmol/L)	−0.03	−0.37	0.70	--	--	--
Phosphorus (mmol/L)	−0.28	−2.98	<0.01*	−0.12	−0.14	0.16
iPTH (pg/ml)	−0.25	−2.62	0.01*	−0.43	−0.49	0.66
rGFR (ml/min/1.73 m^2^)	0.15	1.49	0.13	--	--	--
Kt/V	−0.15	−1.57	0.12	--	--	--
hsCRP(mg/dl)	0.19	1.96	0.05	--	--	--
NLR	0.43	4.74	<0.01*	0.33	3.91	<0.01*

*MAP* mean arterial pressure, *UF* ultrafiltation, *GFR* glomerular filtration rate, *hsCRP* high-sensitive C-reactive protein, *WBC* white blood count, *baPWV* brachial-ankle pulse wave velocity, *NLR* neutrophil-lymphocyte ratio;*p < 0.05

## Discussion

This study revealed that NLR was higher in the low baPWV group than that in the high baPWV group. Moreover, it showed that NRL, a new inflammatory marker, was associated with arterial stiffness in patients with PD. Multiple linear regression analysis showed that NLR was an independent factor for increased baPWV.

Compared to general population, CKD patients have increased arterial stiffness [10]. Arterial stiffness plays a key role in the pathophysiology of the cardiovascular disease. baPWV is a commonly applied non-invasive technique to measure arterial stiffness [11]. Accumulated evidence has indicated that baPWV is an independent predictor of cardiovascular outcomes in patients with CKD [12, 13]. In our study the average level of baPWV was higher in patients with AVD history than those without AVD history. Recent studies have emphasized the importance of inflammation in the pathogenesis of arterial stiffness. Duprez et al. have demonstrated a significant correlation between arterial stiffness and hsCRP [14]. Also, previous studies suggested that reduction in inflammation can decrease arterial stiffness [15].

Recent studies have shown that a potential association between NLR and baPWV in patients with type 1 diabetes [16], osteoporosis [17] or psoriasis [18]. Our study was also in accordance with these studies. The study is the first to show that serum NLR was positively and independently correlated with baPWV in adult PD patients, even after adjustment for various confounders. Moreover, NLR was superior to hsCRP in association with PWV. These results demonstrated that inflammation plays a crucial role in the development of arterial stiffness among patients with PD [19].

The NLR reflects both neutrophil and lymphocyte counts. Our results showed that neutrophil counts were similar in both groups with high or low baPWV, but lymphocyte counts showed significant decrease and NLR showed significant increase in the high group compared to the low baPWV. Thus, increased NLR are more likely due to decreased absolute lymphocyte counts rather than increased neutrophil counts in our study. This is consistent with the study by Solak et al. [20]. The previous study has shown that NLR is superior to these two leukocyte parameters, the most powerful simple leukocyte count indicators [21]. Compared to these two count indicators, it is more stable and less affected by acute conditions which change one of the individual cell counts.

Patients with PD were in a low graded chronic inflammation state. WBC subtypes play the role in different ways to promote atherosclerosis. Increasing evidence has shown that neutrophil directly acts as a mediator of tissue destruction to the arterial wall in inflammation conditions [22]. Lymphopenia may be caused by lymphocyte apoptosis in atherosclerotic lesions, which gradually increases with atherosclerotic burden [23]. Moreover, several studies showed a relationship between low lymphocyte count and malnutrition [24, 25]. Thus, the combination of elevated neutrophils (nonspecific inflammation) and decreased levels of lymphocytes (regulatory component) into a single composite marker may provide additional information to interpret the pathogenesis of arterial stiffness [26]. However, there is an ongoing debate whether NLR is merely a surrogate marker of understanding inflammation, or whether it confers an increased risk per se [20]. More researches are warranted to investigate further.

There are three main limitations in this study. Firstly, our study population is just from a single center, which cannot represent the general population. Secondly, due to the nature of this cross-section study design, we cannot draw a cause-and-effect relationship. Thirdly, as there is no healthy control, it is hard to gain the comparison of baPWV and NLR between patients with PD and healthy persons.

## Conclusion

In conclusion, our results showed that NLR was independently associated with arterial stiffness. It is stable and ready to evaluate NLR and baPWV to confirm inflammation and subclinical atherosclerosis in patients with PD. However, further prospective studies are needed to confirm a cause-and-effect relationship between NLR and baPWV and to investigate whether anti-inflammatory treatment could improve arterial stiffness in patients with PD.

## Additional file

Additional file 1: Raw data of the study. (XLS 46 kb)

## Abbreviations

AVD: Atherosclerotic vascular disease
baPWV: Brachial-ankle pulse wave velocity
BMI: Body mass index
CKD: Chronic kidney disease
ESRD: End stage renal disease
GFR: Glomerular filtration ratev
hsCRP: Highly sensitive C-reactive protein
iPTH: Intact parathyroid hormone
MAP: Mean arterial pressure
MIAC: Malnutrition-inflammation-atherosclerosis and calcification
NLR: Neutrophil-lymphocyte ratio
PD: Peritoneal dialysis
RRF: Residual renal function
WBC: White blood cell
